# Horn size is linked to Sertoli cell efficiency and sperm size homogeneity during sexual development in common eland (*Taurotragus oryx*)

**DOI:** 10.3389/fcell.2024.1421634

**Published:** 2024-08-20

**Authors:** Eliana Pintus, Radim Kotrba, José Luis Ros-Santaella

**Affiliations:** ^1^ Department of Veterinary Sciences, Faculty of Agrobiology, Food and Natural Resources, Czech University of Life Sciences Prague, Prague, Czechia; ^2^ Department of Animal Science and Food Processing, Faculty of Tropical AgriSciences, Czech University of Life Sciences Prague, Prague, Czechia

**Keywords:** male fitness, resource allocation, secondary sexual characters, sexual selection, sperm morphometry, spermatogenic efficiency

## Abstract

**Background:** In polygynous species, the development of secondary sexual characters is usually decisive for male reproductive success. However, our understanding about the links between the growth of these traits and reproductive efficiency is still elusive. Most research efforts in this topic have been also focused on adult males, although the development of some secondary sexual characters, like bovid horns, typically starts after birth, continues during the puberty and in some species, such as the common eland, slows or even stops during adulthood. In this study, we investigated the relationships between horn size and testicular function during sexual development in common elands using a comprehensive approach that considers both spermatogenic and sperm parameters.

**Methods:** Twenty-two non-sexually mature common elands were used for the present study. Horn size, body mass, testes mass, and gonadosomatic index were assessed. Spermatogenic activity was determined by cytological and histological analyses. Sperm concentration, morphology, morphometry, and intramale variation in sperm size were evaluated on epididymal sperm samples. Cluster analysis was performed to explore the influence of age on relationships between horn size and reproductive function.

**Results:** We found that bigger horns are associated with increased Sertoli cell efficiency and reduced intramale variation in sperm size. Both parameters were not related to one another while they have shown to be associated with enhanced sperm quality in ungulates. Moreover, horn size was positively linked to the testis mass, sperm concentration, and testicular investment in the seminiferous epithelium. Spiral length and basal circumference were the horn traits most strongly correlated with spermatogenic and sperm parameters as well as those responsible for the sexual dimorphism in this species. Cluster analysis rendered two groups: the first one including males ≤30 months old, while the second one those >30 months old. Horn development and reproductive function were still correlated within age groups, with the strongest relationship found between horn size and sperm size homogeneity in males >30 months old.

**Conclusion:** Taken together, our results indicate that horn size can be regarded as a good index of male reproductive potential during sexual development and provide insights into the role of secondary sexual characters in sexual selection dynamics.

## 1 Introduction

In sexually dimorphic and polygynous species, male secondary sexual traits are regarded as the hallmark of sexual selection as their expression may influence mating success rendering males more successful in male-male contests (e.g., weapons like tusks, antlers, and horns) and/or more attractive to females (e.g., ornaments like plumage/fur color and manes) (Snook et al., 2013; Simmons et al., 2017). The question on whether the investment in secondary sexual traits growth might predict male fertility has been a matter of a vibrant debate over the last decades. Several studies have explored the relationship between male secondary sexual characters and sperm traits in a variety of taxa (e.g., crustaceans: Paschoal and Zara, 2022; insects: Rogers et al., 2008; fish: Kekäläinen et al., 2014; amphibians: Doyle, 2011; birds: Navara et al., 2012; mammals: Malo et al., 2005a), often finding mixed or weak empirical evidence (Mautz et al., 2013). An aspect to be considered is that no study has so far explored the relationship between spermatogenic function and the morphology of secondary sexual characters, inasmuch testis size and sperm quality are commonly regarded as the main determinant of sperm production and competitiveness, respectively. To fill this gap of knowledge, Ramm and Schärer (2014) advocate a more comprehensive approach that, over testis size, also considers the testicular architecture, spermatogenic cell organization and their hierarchical relationships. Testis mass is indeed a vague measure of male investment in the seminiferous epithelium as it also includes connective tissue, smooth muscle, nerves, blood and lymphatic vessels with the fluids herein. Another aspect that has been attracting the attention of evolutionary studies entails the role of intramale variation in sperm size, which has been mostly explored in a small number of mammalian species (i.e., rodents: Šandera et al., 2013; Varea-Sánchez et al., 2014). To date, only one study has investigated the implication of intramale variation in sperm size in an ungulate species, the red deer (Ros-Santaella et al., 2015). In this study, a reduced coefficient of variation (CV) in sperm size was associated with greater testes mass, sperm velocity and normal morphology, but it is still unknown whether the homogeneity in the sperm size is associated with the development of secondary sexual characters. Additionally, most research efforts exploring the relationships between pre- and post-copulatory traits in mammals have been so far focused on sexually mature males (Ferrandiz-Rovira et al., 2014; Dines et al., 2015), even though the development of secondary sexual characters in many species (e.g., some bovids) starts early in the male lifetime and sometimes even do not appreciably grow during adulthood (Geist, 1966).

In several bovids and cervids, horns and antlers are one of the most diverse and elaborate male secondary sexual characters. In addition to their role as sexual traits, horns and antlers play a major role as weapons against predators and body temperature regulators (Picard et al., 1999; Bro-Jørgensen, 2007). Previous studies have shown that horn/antler size and shape in male ungulates are associated with fighting behavior (Lundrigan, 1996; Caro, 2003), major histocompatibility complex (MHC) traits (Ditchkoff et al., 2001), parasite abundance (Ezenwa and Jolles, 2008), testis size (Malo et al., 2005a), sperm motility and velocity (Malo et al., 2005b; Santiago-Moreno et al., 2007), and reproductive success (Kruuk et al., 2002; Preston et al., 2003; Willisch et al., 2015). In a comparative study in ungulates, Ferrandiz-Rovira et al. (2014) found that longer weapons were significantly associated with shorter sperm cells, but there was no association between weapons length and testes mass (but see also Lüpold et al., 2015). In addition to their size, also fluctuating asymmetry in bilateral paired organs, like horns, can be used as an index of developmental stability (Benítez et al., 2020). In ungulates, fluctuating asymmetry in male secondary sexual traits has been related to poor ejaculate quality, at least in captive populations (Roldan et al., 1998). Surprisingly, the links between horn size/asymmetry and testicular function are still largely unknown in ungulates.

The aim of this study was to explore the relationships between horn morphology (i.e., size and asymmetry) and reproductive competence during the sexual development of male common eland (*Taurotragus oryx*). The common eland is one of the largest antelope species native to the southern and eastern Africa. This non-territorial and sexually dimorphic species is currently classified as “least concern” by the IUCN, with a stable population of ∼100,000 individuals (IUCN, 2024). The age of sexual maturity is estimated to be around 2.5 years in females and 4 years in males (Pappas, 2002). Elands can reproduce at any time of the year, although in wild conditions breeding and calving season peak might occur during the rainy season (Pappas, 2002; Pennigton, 2009). Both sexes have spiral horns, which are shorter, thicker, and have tighter and more pronounced spirals in males than in females (Pappas, 2002). Final horn length is achieved relatively early during male development and do not appreciable grow after reaching sexual maturity (Jeffery and Hanks, 1981). For this reason, sexual development can represent a crucial phase of the male lifetime in which a link between primary and secondary characters is established. The understanding of the role played by horn morphology during the male sexual development can reveal early important traits of individual fitness that might be determinant for reproductive success during adulthood.

## 2 Methods

### 2.1 Animal management and experimental design

**FIGURE 1:**
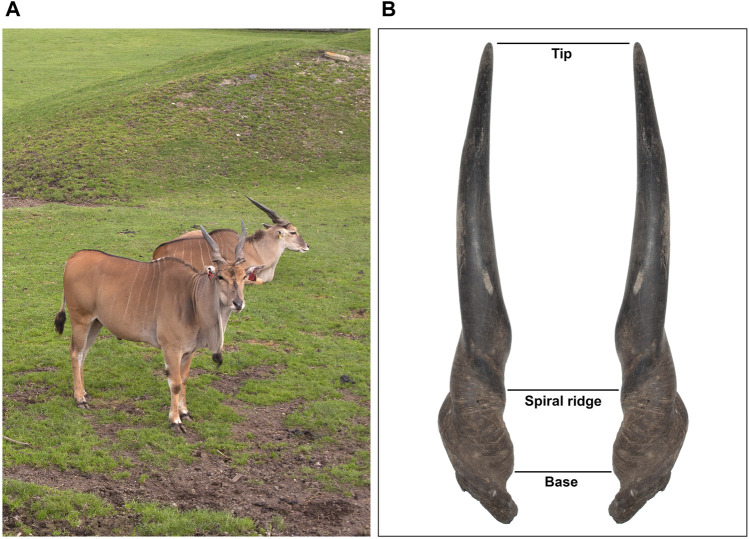
Post-pubertal males and horn measurements in common eland. **(A)** Post-pubertal male common elands. **(B)** Specular image that representatively shows the points of reference for horn size measurements: horn length (from the front base and straight up to the tip), spiral length (from the front base following the spiral ridge to the point where the latter is not pronounced and then straight up to the tip), and basal circumference.

From September 2013 to December 2017, 22 male common elands (age: 15–44 months old; Figure 1A) were slaughtered at the farm of the Czech University of Life Sciences Prague (Lány, Czech Republic). The farm is accredited as research facility according to European and Czech laws for ethical use of animals in research (permits no. 58176/2013-MZE-17214 and no. 63479/2016-MZE-17214). Slaughter of males is part of farm regular production and management to reduce the number of animals due to overwintering capacity. All individuals were born in captivity, individually identified by ear tags since birth, and bred under the same environmental conditions. They represented the seventh captive generation after their import from East Africa from 1969 to 1972 (Vágner, 1974). Genetic diversity of the herd has been maintained through exchanges of individuals from zoological gardens. All eland males were group-housed in straw bedded pens in a barn together with the rest of the herd of maximum of 50 animals, which was separated usually into two groups based on reproductive state of adult females and time of year. All animals were fed with mixed diets *ad libitum* consisted of corn silage (60%), lucerne haylage (30%), meadow hay (7%), and barley straw (3%). This mixture contained 16.6% of crude protein and 16.2% of crude fiber. From April to November, they had access to 2.5 ha of paddock with grass to enhance contact between animals and received up to 1 kg/individual/day of barley grain. The health of animals was randomly checked by veterinarian inspection and monitored on yearly basis. The animals were slaughtered, exsanguinated, eviscerated at the farm, and transported to the abattoir of the Institute of Animal Science in Prague for further processing (see more in Bartoň et al., 2014). All slaughter process was carried out under the supervision of a state veterinarian according to EU and national legislation and conditions for farm animals (slaughter permits no. SVS/WS22/2012-KVSS and no. SVS/2015/077267-S). Testes (within the scrotum) were removed directly after the slaughter, stored in sealed plastic bags, labelled according to individual identification code, and transported at room temperature to the laboratory for further processing. Testicular and sperm samples were collected and processed within approximately 2 h after the death of the animals.

### 2.2 Biometrics and blood testosterone levels

Body mass was determined using tensometric scale (EC 2000, True-Test Limited, Auckland, New Zealand) to the nearest 0.5 kg. Horn size was determined by flexible tape measure to the nearest 0.5 cm. Horn size measurements were determined as it follows: horn length (from the front base and straight up to the tip), spiral length (from the front base following the spiral ridge to the point where it is not pronounced and then straight up to the tip), and basal circumference (Figure 1B). All horn measurements were taken by the same trained observer (RK) and averaged from the left and right sides. Horn asymmetry was calculated as the signed difference between right and left sides of each trait (Palmer, 1994). Horn size was not adjusted for body size as scaling relationship between organs typically occurs during ontogenetic growth (Vea and Shingleton, 2021). In comparative biology, recent studies have also questioned traditional methods for body-size adjustment as they i) do not adequately separate the effects of body size from those of other biological and ecological factors on a specific phenotypic trait (Glazier, 2022) and ii) can spuriously change the sign of regression coefficients compared to the original values, which could lead to inferential biases in biological studies (Rogell et al., 2019). Testosterone levels were assessed from blood samples collected from the jugular veins and carotid arteries into ethylenediaminetetraacetic acid vials. At the laboratory, the samples were immediately centrifuged at 3,500 × *g* for 20 min at 4°C. After that, blood plasma was transferred into a new vial and stored at −80°C till analyses. Testosterone levels were assessed in duplicate by an enzyme immunoassay with a double-antibody technique and expressed as ng/mL (Roelants et al., 2002). Because of logistic issues, blood testosterone levels were determined only during the first phase of the study (*N* = 13).

### 2.3 Testes mass and spermatogenic function

**FIGURE 2:**
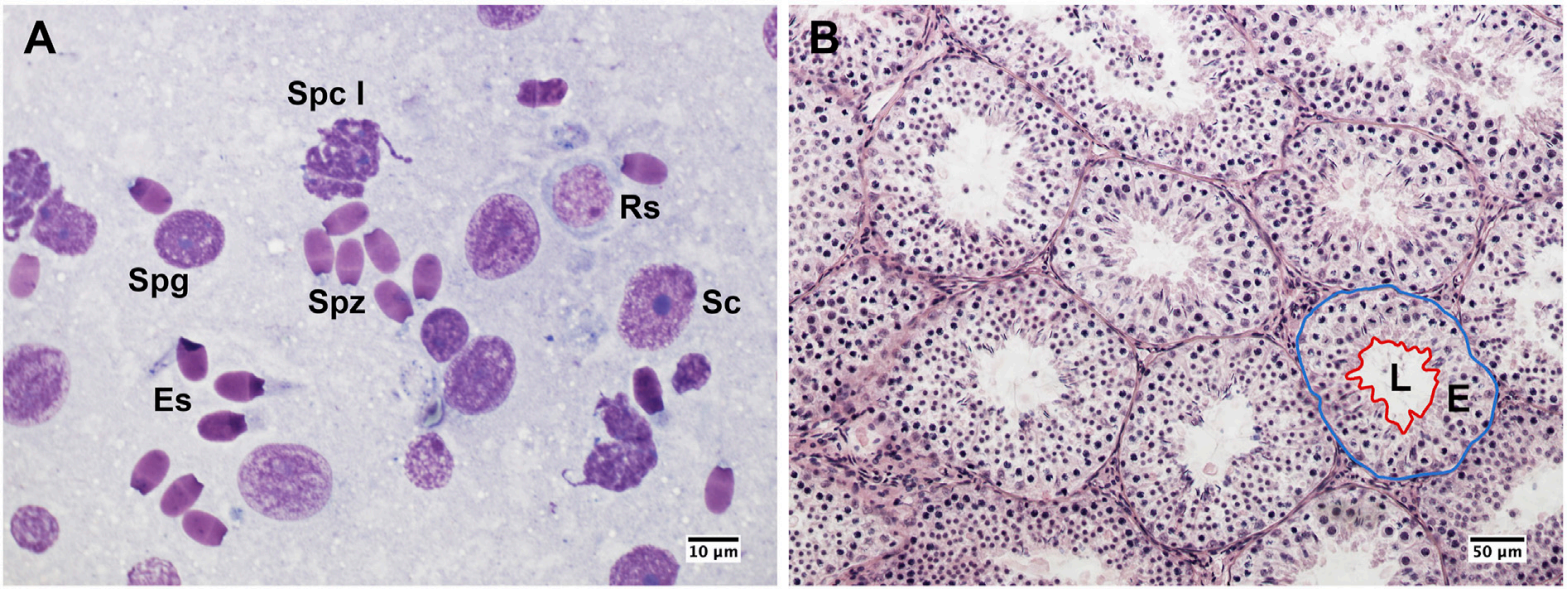
Testicular cytology and histology from post-pubertal common eland. **(A)** Germ cells and Sertoli cells from a cytological smear. Sc: Sertoli cell; Spg: spermatogonium; Spc I: Primary spermatocyte; Rs: round spermatid; Es: elongated spermatid; Spz: spermatozoon. **(B)** Histological section of seminiferous tubules that shows a representative example of seminiferous tubule measurements: the blue line indicates the area of the seminiferous tubule, whereas the red line indicates the epithelial (E) and luminal (L) areas.

Testes mass was recorded to the nearest 0.1 g using an electronic balance (EK-600G, LTD, Japan). The gonadosomatic index (GSI) was calculated as the relative proportion of testes mass to the body mass. Cytological samples were collected from each testis using the fine needle aspiration technique, which has proven to be a reliable method for the assessment of testicular function in ungulates both under physiological and pathological conditions (Pintus et al., 2014; Pintus et al., 2015a). Testicular smears were stained with Hemacolor (Merck, Darmstadt, Germany) and evaluated under a ×100 objective using bright-field microscopy (Nikon Eclipse E600, Nikon, Tokyo, Japan). Assessment of germ cell proportions and spermatogenic indices were determined on at least 200 Sertoli and spermatogenic cells per testis (Figure 2A). Then, testicular indices were assessed as follows: i) the Sertoli cell index (SEI), which is the percentage of Sertoli cells per total germ cells and estimates the spermatogenic activity; ii) the spermatozoa-Sertoli cell index (SSEI), which is the number of spermatozoa per Sertoli cell; iii) the meiotic index (MI), which is the ratio of round spermatids to primary spermatocytes and estimates the germ cell loss during meiosis; iv) the ratio of elongated spermatids to round spermatids (ES/RS), which estimates the germ cell loss during the post-meiotic phase; v) the ratio of elongated spermatids to total germ cells (ES/GC), which estimates the overall germ cell loss during spermatogenesis; vi) the ratio of round spermatids to Sertoli cells (RS/SC), vii) the ratio of elongated spermatids to Sertoli cells (ES/SC), which both estimate the Sertoli cell function; and viii) the ratio of total germ cells to Sertoli cells (GC/SC) that estimates the Sertoli cell workload capacity (Ros-Santaella et al., 2019). Samples for testicular histology were collected and processed as previously described (Pintus et al., 2015a). Briefly, a fragment of approximately 1 cm^3^ was isolated from the equatorial region of each testis, fixed in modified Davidson’s solution (30% formaldehyde, 15% ethanol, 5% glacial acetic) for 24–48 h, then stored in 70% ethanol until analysis. Then, the samples were embedded in paraffin, cut into 4 µm sections, and stained with hematoxylin and eosin. To evaluate the morphology of the seminiferous tubules, 25 roundish cross-sections of the seminiferous tubules were photographed per each testis using a high-resolution camera (Digital Sight DSFi1, Nikon, Tokyo, Japan) under a ×20 objective. Then, the area of the seminiferous tubule, lumen, and epithelium were assessed using ImageJ software (National Institutes of Health, Bethesda, MD, United States) (Figure 2B). The proportion of the seminiferous epithelium to the total tubular area was then calculated. Values from cytological and histological analyses were averaged from the left and right testes. Because of suboptimal sample quality or reduced smear cellularity, testicular cytology and histology were not performed in one and two males, respectively. All measurements were taken by the same trained observer (EP).

### 2.4 Sperm concentration, morphology, and morphometry

**FIGURE 3:**
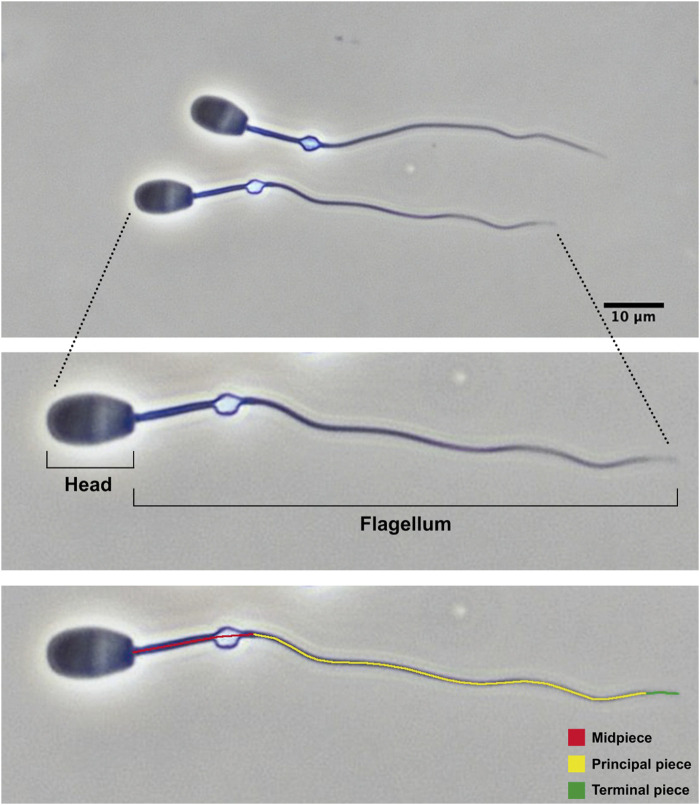
Epididymal spermatozoa from post-pubertal common eland and their main structures.

Sperm samples were collected from the epididymal caudae using a sterile surgical blade and fixed into 0.5 mL phosphate-buffered saline solution supplemented with 2% glutaraldehyde. Sperm concentration was determined using a Bürker chamber. The percentage of morphologically normal spermatozoa was determined after evaluating 200 spermatozoa under phase-contrast microscopy (×40 objective). Sperm morphometry was assessed as previously described (Ros-Santaella et al., 2014; Ros-Santaella et al., 2015). Briefly, sperm pictures were taken with high-resolution camera under phase-contrast microscopy (×40 objective). The following sperm traits were determined using the ImageJ software: head width, head length, midpiece length, and flagellum length. From these measurements, other morphometric parameters were calculated such as head area, head perimeter, head ellipticity (head length/head width), total sperm length, and principal plus terminal piece length. Twenty-five spermatozoa with normal morphology (i.e., without head or flagellum abnormalities and without proximal cytoplasmic droplet) were measured per male. For each parameter, the intramale coefficient of variation (CV) was calculated as standard deviation/mean×100. The main structures of common eland spermatozoa with normal morphology are shown in Figure 3. Sperm morphology and morphometry could not be assessed in five samples because of their low sperm concentration or smear quality. All measurements were taken by the same trained observer (JR-S).

### 2.5 Statistical analysis

Statistical analyses were performed using the SPSS 20.0 statistical software (IBM Inc., Chicago, IL, United States). The Shapiro-Wilk test was used to check for the normal distribution of data. Data that were not normally distributed were log-transformed. Non-parametric tests were applied to data that were not normally distributed after log transformation. Principal component analysis was used to reduce several closely related variables (i.e., horn size/asymmetry, Sertoli cell efficiency, and intramale variation in sperm head size) into a smaller subset that better summarizes the original data. The Bartlett sphericity and Keiser-Meyer-Olkin (KMO) tests for sampling adequacy were applied to test the suitability of data for the principal component analysis. The principal components (PCs) with Bartlett sphericity test’s *p*-value higher than 0.05 and KMO test lower than 0.5 were considered inappropriate (Budaev, 2010). To determine the influence of age on horn and reproductive relationships, two-step cluster analysis was applied using age of males expressed in months as a continuous variable. The number of clusters was automatically determined using the Euclidean distance measure and the Schwarz’s Bayesian criterion. Subsequently, the number of clusters previously obtained was used to set up the K-Means cluster analysis by using the iteration and classification method. Unless otherwise specified, two-tailed Pearson correlations were used when data were normally distributed, otherwise two-tailed Spearman correlations. Correlation analyses were not corrected for multiplicity because, while correction methods decrease the probability of Type I error, they increase the probability of Type II error (Streiner, 2015). Therefore, although the statistical significance was set at *p* < 0.05, *p* values close to 0.05 must be cautiously considered. Data are shown as the mean ± SE.

## 3 Results

Descriptive statistics of biometrics, testicular, and sperm parameters of male common elands are shown in Tables 1, 2. On average, horn size was around 55 cm in length and 26 cm at basal circumference, while the spiral length was 68 cm. The signed asymmetry between the right and left sides of each horn trait was: −0.68 ± 0.36 cm for horn length, −0.64 ± 0.46 cm for spiral length, and −0.14 ± 0.19 cm for basal circumference. Except one male in which testicular cytology could not be assessed although spermatozoa were present in the epididymal caudae, the spermatogenesis was complete in all individuals, which indicate that males did already reach puberty. Cytological and histological analyses showed that spermatozoa represent around one-fifth of spermatogenic cell population, with over 80% of the tubular area filled by the seminiferous epithelium. Among spermatogenic cells, the round spermatids were the most abundant, while secondary spermatocytes were the scarcest. On average, over 700×10^6^ spermatozoa/mL were collected from the epididymal caudae, with two-thirds of them being morphologically normal. The proportion of each sperm structure in relation to the total sperm length was: head length, 13.45%; midpiece length, 20.21%; and principal piece plus terminal piece length, 66.34%. Overall, size variation in sperm parameters was below 5%, relatively higher CV values were found in the sperm head than in the flagellum (Table 2).

**TABLE 1:** Descriptive statistics of biometrics and testicular parameters in post-pubertal common eland.

	Mean ± SE	*N*
*Biometrics and blood testosterone levels*		
Body mass (kg)	273.25 ± 16.23	22
Horn length (cm)	55.23 ± 1.07	22
Horn spiral length (cm)	67.82 ± 1.50	22
Horn basal circumference (cm)	25.89 ± 0.29	22
Testes mass (g)	123.43 ± 7.28	22
GSI (%)	0.05 ± 0.00	22
Blood testosterone levels (ng/mL)	0.37 ± 0.06	13
*Spermatogenic cells*		
Spermatogonia (%)	2.25 ± 0.25	21
Primary spermatocytes (%)	22.65 ± 0.83	21
Secondary spermatocytes (%)	0.88 ± 0.10	21
Round spermatids (%)	37.51 ± 0.69	21
Elongated spermatids (%)	16.76 ± 0.67	21
Spermatozoa (SI, %)	19.95 ± 0.76	21
*Spermatogenic indices*		
SEI (%)	11.44 ± 1.96	21
SSEI	2.51 ± 0.33	21
MI	1.72 ± 0.08	21
ES/RS	0.46 ± 0.02	21
ES/GC	0.17 ± 0.01	21
RS/SC	4.74 ± 0.68	21
ES/SC	2.05 ± 0.25	21
GC/SC	12.37 ± 1.61	21
*Testicular architecture*		
Area seminiferous tubule (µm^2^)	26,520.26 ± 1,050.76	20
Area seminiferous epithelium (µm^2^)	22,033.46 ± 936.32	20
Area seminiferous lumen (µm^2^)	4,486.80 ± 219.69	20
Proportion of the seminiferous epithelium to the tubular area (%)	83.07 ± 0.82	20

GSI, gonadosomatic index; SI, spermatic index; SEI, Sertoli cell index; SSEI, spermatozoa-Sertoli cell index; MI, meiotic index; ES/RS, ratio of elongated spermatids to round spermatids; ES/GC, ratio of elongated spermatids to germ cells; RS/SC, ratio of round spermatids to Sertoli cells; ES/SC, ratio of elongated spermatids to Sertoli cells; GC/SC, ratio of germ cells to Sertoli cells.

**TABLE 2:** Descriptive statistics of sperm parameters in post-pubertal common eland.

	Mean ± SE	*N*
*Sperm number and morphology*		
Sperm concentration (10^6^/mL)	755.32 ± 100.76	21
Normal sperm (%)	68.79 ± 7.01	17
*Sperm size*		
Head width (μm)	5.92 ± 0.04	17
Head length (μm)	9.66 ± 0.07	17
Head perimeter (μm)	24.84 ± 0.13	17
Head area (μm^2^)	44.95 ± 0.44	17
Head ellipticity	1.63 ± 0.02	17
Midpiece length (μm)	14.52 ± 0.10	17
Principal plus terminal piece length (μm)	47.66 ± 0.38	17
Flagellum length (μm)	62.17 ± 0.42	17
Sperm length (μm)	71.84 ± 0.43	17
*Intramale CV in sperm size*		
Head width (%)	3.29 ± 0.20	17
Head length (%)	2.85 ± 0.15	17
Head perimeter (%)	2.21 ± 0.10	17
Head area (%)	4.35 ± 0.21	17
Head ellipticity (%)	4.46 ± 0.20	17
Midpiece length (%)	2.68 ± 0.16	17
Principal plus terminal piece length (%)	2.07 ± 0.08	17
Flagellum length (%)	1.57 ± 0.07	17
Sperm length (%)	1.49 ± 0.06	17

CV, coefficient of variation.

### 3.1 Principal component analysis of horn size and asymmetry

A principal component analysis was performed using the averaged values of the left and right horn measurements to obtain smaller subset of variables that summarizes the horn size. We obtained a single principal component (PC1, horn size) that overall explained 75.89% of the total variance (Keiser-Meyer-Olkin, KMO, measure of sampling adequacy = 0.520; Bartlett’s test of sphericity: approx. χ^2^ = 44.41, df = 3, *p* < 0.0001; Table 3) and showed normal distribution (Shapiro-Wilk test, *p* = 0.872). Another principal component analysis was performed using the signed difference between the right and left horn measurement to obtain a smaller subset of variables that explain horn asymmetry. We obtained a single principal component (PC1, horn asymmetry) that overall explained 60.29% of the total variance (KMO measure of sampling adequacy = 0.516; Bartlett’s test of sphericity: approx. χ^2^ = 16.30, df = 3, *p* = 0.001; Table 3). The PC1 of horn asymmetry shows normal distribution (Shapiro-Wilk test, *p* = 0.552) around a mean value close to zero (Skewness = −0.554 ± 0.491 and Kurtosis = 0.496 ± 0.953), which is indicative of fluctuating asymmetry.

**TABLE 3:** Principal component analysis of horn size and asymmetry in post-pubertal common eland.

	PC1	*p*
*Horn size*		
Horn length	0.916	<0.001
Spiral length	0.967	<0.001
Basal circumference	0.709	<0.001
Eigenvalue	2.28	
Variance explained (%)	75.89	
*Horn asymmetry*		
Horn length	0.923	<0.01
Spiral length	0.909	<0.01
Basal circumference	0.361	<0.05
Eigenvalue	1.81	
Variance explained (%)	60.29	

PC, Principal component. Horn asymmetry was calculated as the signed value of the difference between right and left sides of each trait.

### 3.2 Correlations between horn size and reproductive function

#### 3.2.1 Correlations of horn size with testicular and spermatogenic parameters

**FIGURE 4:**
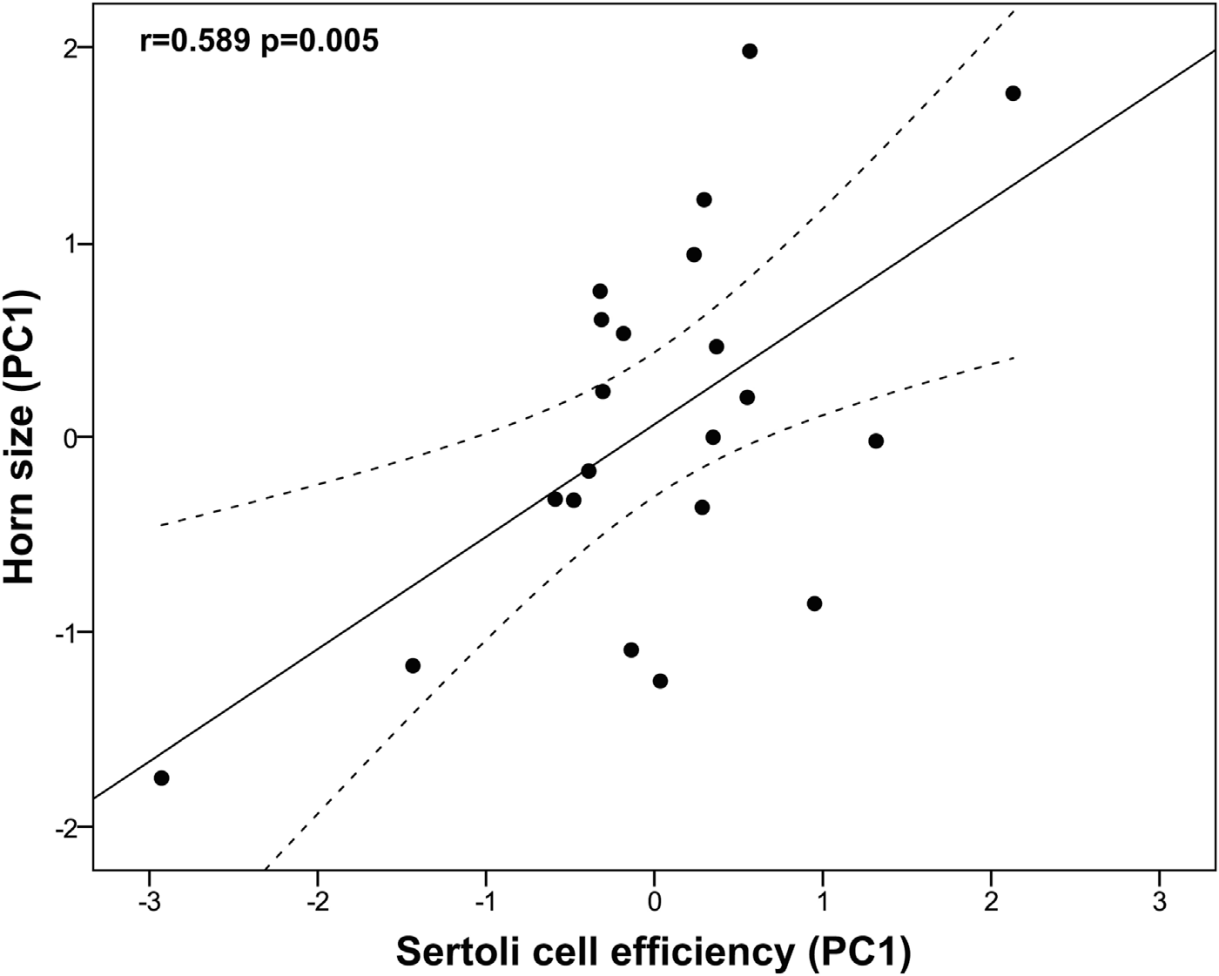
Relationship between horn size and Sertoli cell efficiency in post-pubertal common eland. Black line represents linear fit line, while dashed lines indicate 95% confidence intervals. PC: Principal component.

We found that horn size was significantly associated with higher testes mass (r = 0.476, *p* = 0.025), while it did not correlate neither with the GSI (log-transformed; r = 0.083, *p* = 0.713) nor with the blood testosterone levels (r = 0.176, *p* = 0.565). Albeit no significant, horn size was positively associated with the body mass (log-transformed; r = 0.421, *p* = 0.051). An unexpected finding was that horn size was not related to the proportion of any spermatogenic cell type, including the spermatic index that represents the proportion of spermatozoa over the total germ cells and estimates the sperm production (r = 0.071, *p* = 0.761). On the other hand, larger horn size was associated with reduced Sertoli cell index (SEI, log-transformed r = −0.590, *p* = 0.005) and increased Sertoli cell function and workload capacity (i.e., spermatozoa-Sertoli cell index, SSEI: r = 0.531, *p* = 0.013; ratio of round spermatids to Sertoli cells, RS/SC: r = 0.540, *p* = 0.012; ratio of elongated spermatids to Sertoli cells, ES/SC: rho = 0.516, *p* = 0.017; ratio of total germ cells to Sertoli cells, GC/SC: r = 0.581, *p* = 0.006, all log-transformed). Among the three descriptors of horn size, spiral length was the one showing the strongest relationship with Sertoli cell function and workload (i.e., spermatozoa-Sertoli cell index, SSEI: r = 0.603, *p* = 0.004; ratio of round spermatids to Sertoli cells, RS/SC: r = 0.610, *p* = 0.003; ratio of elongated spermatids to Sertoli cells, ES/SC: rho = 0.527, *p* = 0.014; ratio of total germ cells to Sertoli cells, GC/SC: r = 0.650, *p* = 0.001, all log-transformed). To corroborate our findings, we performed a principal component analysis of Sertoli cell indices because of the high correlation among them. We obtained a single principal component (PC1, Sertoli cell efficiency) that explained 96.74% of the total variance (KMO measure of sampling adequacy = 0.803; Bartlett’s test of sphericity: approx. χ^2^ = 268.75, df = 10, *p* < 0.0001; Table 4) and showed normal distribution (Shapiro-Wilk test, *p* = 0.055). Our findings confirm that larger horn size was associated with increased Sertoli cell efficiency (r = 0.589, *p* = 0.005, Figure 4).

**TABLE 4:** Principal component analysis of Sertoli cell efficiency and intramale variation in sperm head size in post-pubertal common eland.

	PC1	*p*
*Sertoli cell efficiency*		
log SEI	−0.997	<0.001
log SSEI	0.968	<0.001
log RS/SC	0.986	<0.001
log ES/SC	0.968	<0.001
log GC/SC	0.998	<0.001
Eigenvalue	4.84	
Variance explained (%)	96.74	
*Intramale variation in sperm head size*		
log head width CV	0.938	<0.001
Head area CV	0.937	<0.001
Head ellipticity CV	0.901	<0.001
Eigenvalue	2.57	
Variance explained (%)	85.64	

CV, coefficient of variation; SEI, Sertoli cell index; SSEI, spermatozoa-Sertoli cell index; RS/SC, ratio of round spermatids to Sertoli cells; ES/SC, ratio of elongated spermatids to Sertoli cells; GC/SC, ratio of germ cells to Sertoli cells. PC, principal component.

Horn size was also positively related to the area of the seminiferous tubule and epithelium (r = 0.462, *p* = 0.040 and r = 0.531, *p* = 0.016, respectively). There was also a significant positive relationship between horn size and the proportion of the tubular area lined by the seminiferous epithelium (rho = 0.456, *p* = 0.043). Among the three descriptors of horn size, basal circumference was the one showing the strongest relationship with the area of seminiferous tubule and epithelium (r = 0.640, *p* = 0.002 and r = 0.629, *p* = 0.003, respectively). We performed a principal component analysis of testicular architecture, which rendered one principal component that explained 77.13% of the total variance. However, because of the low value of KMO measure of sampling adequacy test (i.e., 0.327), the principal component was rejected.

#### 3.2.2 Correlations of horn size with sperm parameters

**FIGURE 5:**
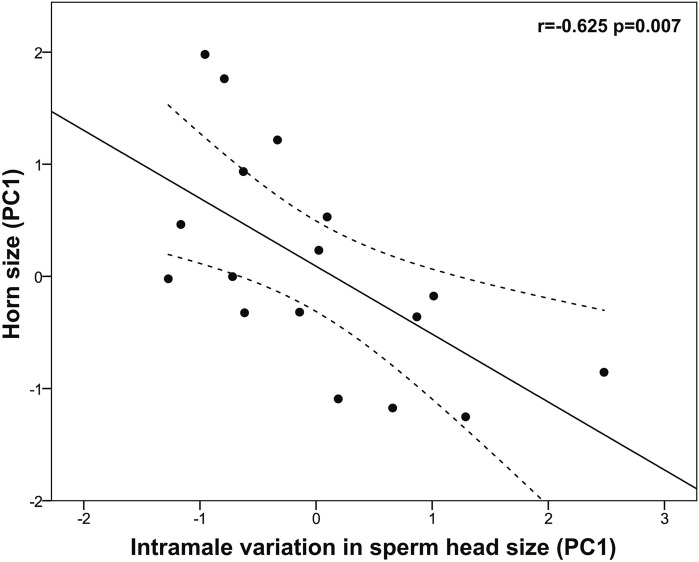
Relationship between horn size and sperm head size homogeneity in post-pubertal common eland. Black line represents linear fit line, while dashed lines indicate 95% confidence intervals. PC: Principal component.

Horn size was positively associated with sperm concentration (r = 0.543, *p* = 0.011) but it did not correlate with either sperm morphology or size (*p* > 0.05). Albeit not significant, large horn size was associated with a reduced midpiece length (r = −0.481, *p* = 0.051). Interestingly, bigger horn size was significantly associated with reduced intramale variation in sperm head size (log-head width CV: r = −0.614, *p* = 0.009; head area CV: r = −0.520, *p* = 0.033) and shape (head ellipticity CV: r = −0.602, *p* = 0.011). Among the three descriptors of horn size, basal circumference was the one showing the strongest relationship with intramale CV of sperm head size and shape (i.e., log-head width CV: r = −0.549, *p* = 0.023; head area CV: r = −0.645, *p* = 0.005; head ellipticity CV: r = −0.664, *p* = 0.004). To corroborate our findings, a principal component analysis of sperm head CV parameters was performed. We obtained a single principal component (PC1, intramale variation in sperm head size) that explained 85.64% of the total variance (KMO measure of sampling adequacy = 0.746; Bartlett’s test of sphericity: approx. χ^2^ = 31.275, df = 3, *p* < 0.0001; Table 4) and showed normal distribution (Shapiro-Wilk test, *p* = 0.234). Our findings support that larger horn size is associated with reduced intramale variation in sperm head size and shape (r = −0.625, *p* = 0.007, Figure 5).

#### 3.2.3 Correlations between spermatogenic and sperm parameters

To exclude collinearity, we checked for correlation between spermatogenic and sperm parameters that have previously shown to be associated with horn size. We found that increased Sertoli cell efficiency was associated with greater testes size (r = 0.652, *p* = 0.001), sperm concentration (r = 0.475, *p* = 0.034), and investment in tubular and epithelial areas of the testicular parenchyma (r = 0.515, *p* = 0.024 and r = 0.587, *p* = 0.008, respectively). Surprisingly, reduced intramale variation in sperm head size was not significantly associated either with testes mass (r = −0.167, *p* = 0.522), sperm concentration (r = −0.391, *p* = 0.121) or Sertoli cell efficiency (r = −0.248, *p* = 0.338). However, low intramale variation in sperm head ellipticity was associated with greater sperm concentration (r = −0.500, *p* = 0.041) and larger areas of the seminiferous tubule and epithelium (r = −0.590, *p* = 0.021 and r = −0.591, *p* = 0.020, respectively).

We also checked the correlations between the remaining spermatogenic and sperm parameters (for simplicity, only those with *p* < 0.01 are shown herein). We found that high Sertoli cell efficiency was associated with smaller sperm head (i.e., head width: r = −0.659, *p* = 0.004; head area: r = −0.678, *p* = 0.003), while high meiotic index was strongly associated with longer sperm length (i.e., principal plus terminal piece length: r = 0.834, *p* < 0.0001; flagellum length: r = 0.822, *p* < 0.0001; total sperm length: r = 0.819, *p* < 0.0001). Sperm concentration was positively associated with tubular and epithelial areas of the testicular parenchyma (r = 0.756, *p* < 0.001 and r = 0.771, *p* < 0.001, respectively). Moreover, high proportion of normal spermatozoa was associated with increased Sertoli cell efficiency (rho = 0.725, *p* < 0.001) and reduced intramale CV in sperm length (flagellum length: rho = -0.635, *p* = 0.006; total sperm length: rho = −0.673, *p* = 0.003).

### 3.3 Correlations between horn asymmetry and reproductive function

We found that horn asymmetry (PC1, horn asymmetry) was not significantly correlated neither with biometric, blood testosterone levels, testicular or sperm parameters (*p* > 0.05).

### 3.4 Effect of age on correlations between horn size and reproductive function

**FIGURE 6:**
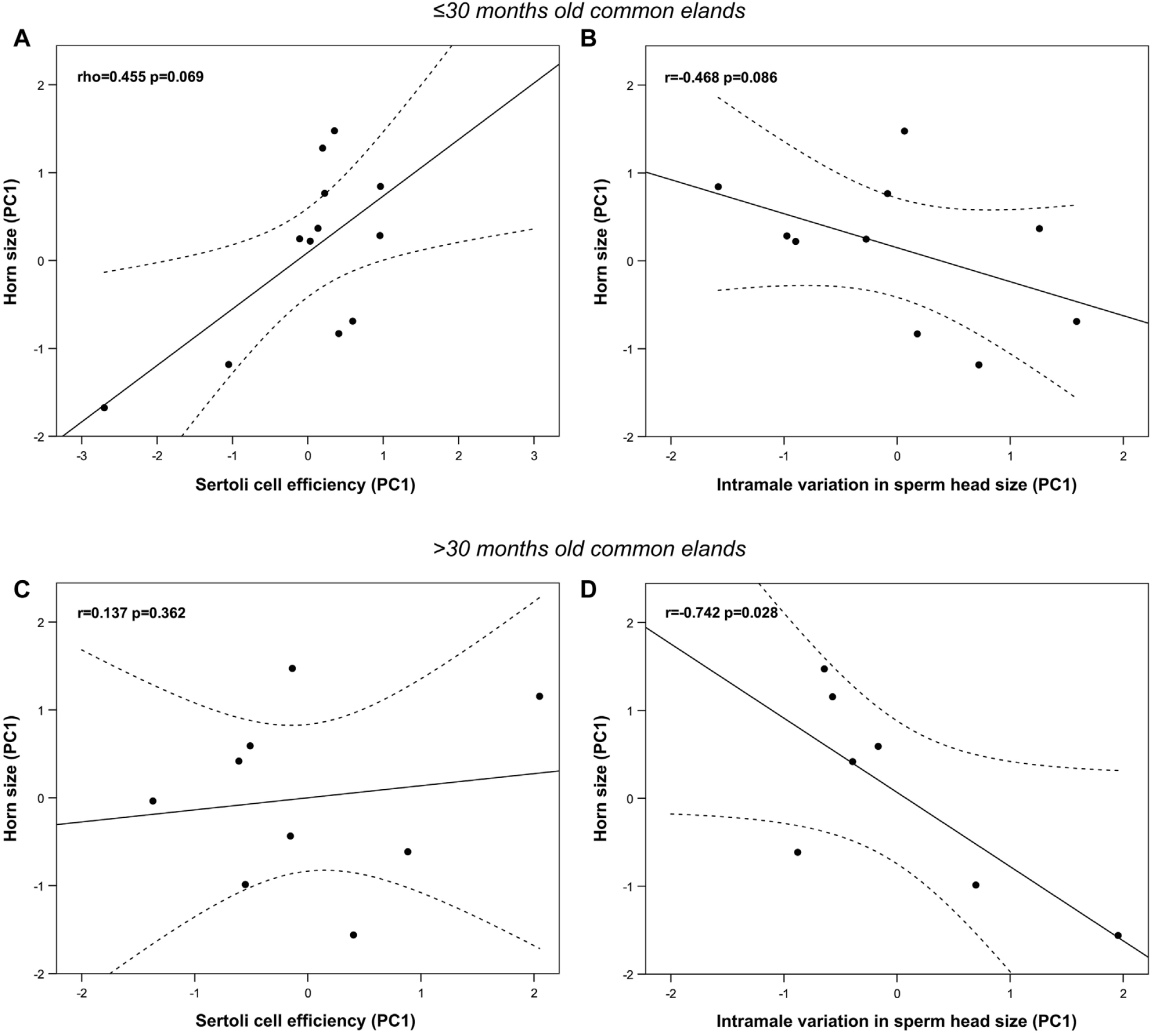
Relationship between horn size, Sertoli cell efficiency, and sperm head size homogeneity in ≤30 months old **(A,B)** and >30 months old **(C,D)** post-pubertal common eland. Black line represents linear fit line, while dashed lines indicate 95% confidence intervals. PC: Principal component.

Cluster analysis rendered two groups of similar sample size: the first one includes males ≤30 months old (n = 13), while the second one those >30 months old (n = 9). Most horn and spermatogenic parameters differed between groups except for horn basal circumference, GSI, blood testosterone levels, percentage of spermatozoa over the total germ cell population, indices of germ cell loss during the post-meiotic phase and whole spermatogenic process (i.e., ES/RS and ES/GC, respectively), and areas of the seminiferous tubule and epithelium (*p* > 0.05, Table 5), to mention but a few. Interestingly, groups did not differ in any epididymal sperm parameter apart from the sperm head width and area that were significantly smaller in elands >30 months old compared to those ≤30 months old (*p* < 0.05, Table 6). New principal component analysis for horn size, Sertoli cell efficiency, and intramale variation in sperm head size were performed for each age group (Supplementary Table S2). Principal component analysis rendered one principal component (PC1) for horn size, Sertoli cell efficiency, and intramale variation in sperm head size in each age group, each of them explaining over 65% of the total variance. The KMO and Bartlett’s test of sphericity were >0.57 and <0.01, respectively, for all PCs except for PC1 of horn size in age group ≤30 months old (i.e., KMO = 0.376). In this group, correlation analysis showed that, albeit no significant, larger horn size was correlated with increased Sertoli cell efficiency and reduced intramale variation in sperm head size (one-tailed rho = 0.455, *p* = 0.069, Figure 6A and one-tailed r = −0.468, *p* = 0.086; Figure 6B, respectively). Although no significant correlation was found with Sertoli cell efficiency in elands >30 months old (*p* > 0.05, Figure 6C), larger horn size was strongly correlated with reduced intramale variation in sperm head size (r = −0.742 and *p* = 0.028, Figure 6D) in this age group.

**TABLE 5:** Comparison of biometrics and testicular parameters in ≤30 months old and >30 months old post-pubertal common elands.

	≤30 months old (Mean ± SE)	>30 months old (Mean ± SE)	*p*
*Biometrics*			
Body mass (kg)	227.50 ± 9.44	339.33 ± 23.93	0.001
Horn length (cm)	53.21 ± 1.19	58.14 ± 1.58	0.019
Horn spiral length (cm)	64.56 ± 1.58	72.53 ± 2.07	0.006
Horn basal circumference (cm)	25.62 ± 0.43	26.28 ± 0.35	0.276
Testes mass (g)	106.09 ± 7.38	148.47 ± 9.48	0.002
GSI (%)	0.05 ± 0.00	0.04 ± 0.00	0.734
Blood testosterone levels (ng/mL)	0.35 ± 0.07	0.41 ± 0.07	0.442
*Spermatogenic cells*			
Spermatogonia (%)	2.00 ± 0.36	2.57 ± 0.30	0.041
Primary spermatocytes (%)	24.13 ± 0.95	20.68 ± 1.23	0.027
Secondary spermatocytes (%)	0.82 ± 0.15	0.95 ± 0.12	0.553
Round spermatids (%)	37.35 ± 0.95	37.71 ± 1.06	0.807
Elongated spermatids (%)	16.39 ± 1.03	17.27 ± 0.78	0.529
Spermatozoa (SI, %)	19.30 ± 1.19	20.83 ± 0.79	0.332
*Spermatogenic indices*			
SEI (%)	14.69 ± 3.11	7.11 ± 0.84	0.002
SSEI	1.76 ± 0.23	3.51 ± 0.57	0.001
MI	1.59 ± 0.08	1.90 ± 0.13	0.044
ES/RS	0.45 ± 0.03	0.47 ± 0.03	0.705
ES/GC	0.16 ± 0.01	0.17 ± 0.01	0.529
RS/SC	3.30 ± 0.36	6.66 ± 1.29	0.004
ES/SC	1.45 ± 0.17	2.85 ± 0.43	0.001
GC/SC	8.79 ± 0.89	17.15 ± 2.94	0.004
*Testicular architecture*			
Area seminiferous tubule (µm^2^)	25,578.69 ± 1,802.32	27,671.06 ± 750.57	0.303
Area seminiferous epithelium (µm^2^)	20,854.38 ± 1,574.66	23,474.56 ± 605.62	0.145
Area seminiferous lumen (µm^2^)	4,724.31 ± 361.58	4,196.50 ± 189.29	0.253
Proportion of the seminiferous epithelium to the tubular area (%)	81.44 ± 1.27	85.06 ± 0.45	0.003

Sample size of ≤30 months old eland group is n = 13 except for blood testosterone levels (n = 10), spermatogenic indices (n = 12), and testicular architecture (n = 11). Sample size of >30 months old eland group is n = 9 except for blood testosterone levels (n = 3). GSI, gonadosomatic index; SI, spermatic index; SEI, Sertoli cell index; SSEI, spermatozoa-Sertoli cell index; MI, meiotic index; ES/RS, ratio of elongated spermatids to round spermatids; ES/GC, ratio of elongated spermatids to germ cells; RS/SC, ratio of round spermatids to Sertoli cells; ES/SC, ratio of elongated spermatids to Sertoli cells; GC/SC, ratio of germ cells to Sertoli cells.

**TABLE 6:** Comparison of sperm parameters in ≤30 months old and >30 months old post-pubertal common elands.

	≤30 months old (Mean ± SE)	>30 months old (Mean ± SE)	*p*
*Sperm number and morphology*			
Sperm concentration (10^6^/mL)	661.09 ± 148.02	908.44 ± 98.48	0.243
Normal sperm (%)	56.70 ± 10.14	86.07 ± 3.66	0.055
*Sperm size*			
Head width (μm)	6.00 ± 0.05	5.81 ± 0.04	0.014
Head length (μm)	9.71 ± 0.09	9.59 ± 0.11	0.397
Head perimeter (μm)	25.04 ± 0.14	24.56 ± 0.21	0.074
Head area (μm^2^)	45.77 ± 0.43	43.78 ± 0.68	0.020
Head ellipticity	1.62 ± 0.02	1.65 ± 0.02	0.318
Midpiece length (μm)	14.57 ± 0.14	14.44 ± 0.15	0.545
Principal plus terminal piece length (μm)	47.19 ± 0.37	48.32 ± 0.71	0.147
Flagellum length (μm)	61.76 ± 0.47	62.76 ± 0.78	0.258
Sperm length (μm)	71.48 ± 0.50	72.35 ± 0.77	0.332
*Intramale CV in sperm size*			
Head width (%)	3.25 ± 0.18	3.33 ± 0.44	0.963
Head length (%)	3.01 ± 0.23	2.63 ± 0.14	0.223
Head perimeter (%)	2.28 ± 0.14	2.11 ± 0.15	0.417
Head area (%)	4.40 ± 0.20	4.28 ± 0.45	0.795
Head ellipticity (%)	4.55 ± 0.24	4.33 ± 0.35	0.598
Midpiece length (%)	2.89 ± 0.20	2.37 ± 0.24	0.112
Principal plus terminal piece length (%)	2.17 ± 0.09	1.92 ± 0.13	0.125
Flagellum length (%)	1.65 ± 0.09	1.47 ± 0.10	0.216
Sperm length (%)	1.56 ± 0.09	1.39 ± 0.06	0.188

Sample size of ≤30 months old eland group is n = 13 except for sperm morphology and morphometry (n = 10). Sample size of >30 months old eland group is n = 8 for sperm concentration and n = 7 for sperm morphology and morphometry. CV, coefficient of variation.

## 4 Discussion

The present study comprehensively explores the relationships between primary and secondary sexual characters during the sexual development of a polygynous ungulate, the common eland. We provide evidence that large horn size is associated with increased testes mass, spermatogenic activity, Sertoli cell efficiency, sperm concentration and reduced intramale variation in sperm size. It is remarkable to point out that the strength of correlations between horn size and Sertoli cell efficiency and between the former and sperm size homogeneity showed r > 0.58 and *p* < 0.01, which provide new insights into the links between primary and secondary sexual characters in a sexually dimorphic mammalian species. After exploring the effect of age using cluster analysis, primary and secondary sexual characters were still correlated within age groups, with the strongest relationship found between horn size and sperm size homogeneity in males approaching sexual maturity. Taken together, our results indicate that, during sexual development, horn size is a good index of reproductive potential in the common eland. It remains however to explore whether increased Sertoli cell efficiency and sperm size homogeneity translate into enhanced male fertility.

Our findings confirm the positive relationship between secondary sexual traits and testis size in ungulates (Malo et al., 2005b) and suggest that such a relationship is mainly supported by the increased Sertoli cell efficiency. Discovered by Enrico Sertoli in 1865, the Sertoli cell represents the only somatic cell found within the seminiferous tubules. The astonishing ability of Sertoli cells to nurse, protect, and support the development of up to five different types of germ cells at any one time make them one of the most complex cells in the body (O’Donnell et al., 2022). The role of Sertoli cells is crucial during the entire male lifetime: during the embryo and fetal development, Sertoli cells promote the sexual differentiation, while during puberty and sexual maturity they coordinate the spermatogenesis and determine the sperm production. Our findings also indicate that greater Sertoli cell efficiency is associated with higher percentage of normal spermatozoa and smaller sperm head size, possibly because of Sertoli cell’s role in phagocytosing abnormal germ cells and residual bodies during spermatogenesis (Oliveira and Alves, 2015). Future studies should be directed towards a deeper understanding of Sertoli cell efficiency using both metabolic and endocrine markers such as lactate production or inhibin levels (Rato et al., 2016; Shah et al., 2021). Taken together, our findings provide further support for the key role of Sertoli cells in determining testis mass, sperm production and quality in ungulates (Pintus et al., 2015b; Ros-Santaella et al., 2019).

Our results show that the investment in secondary sexual characters’ growth is not linked to sperm size but rather to the homogeneity in sperm cell dimension. Findings from comparative studies in this taxonomic group have been so far elusive with both negative (Ferrandiz-Rovira et al., 2014) or no (Lüpold et al., 2015) relationships between horn/antler development and sperm size. Under this perspective, our findings highlight the relevance of intraspecific studies as they may provide a better understanding of patterns observed across animal species and reveal the signatures of selection (Kleven et al., 2008). In red deer, for instance, reduced intramale variation in sperm size is associated with enhanced sperm motility and normal sperm morphology (Ros-Santaella et al., 2015), which predict male fertility in this species (Malo et al., 2005a). In agreement with a previous study (Ros-Santaella et al., 2015), our findings also confirm that normal sperm morphology is associated with reduced intramale variation in sperm length. A smaller within-male CV in sperm size has also been associated with the intensity of sperm competition and testicular investment (Šandera et al., 2013; Ros-Santaella et al., 2015). The lack of significant relationships between intramale variation in sperm size and spermatogenic function in the common elands raise the possibility that other factors, rather than spermatogenesis *per se*, might play a role in the production of a more uniform sperm population. For instance, in the male great tits (*Parus major*), within-male variation in sperm length is associated with a health-related (hematological) trait but not with male ornaments (Svobodová et al., 2018). Beside the cytological and histological assessments provided in this study, analysis of molecular markers and signaling pathways that control germ cell proliferation, differentiation, and apoptosis may contribute to elucidate the mechanisms underlying the relationships between spermatogenic function and sperm traits such as, for instance, the strong positive correlation between the meiotic index and the sperm length found in this study. Another factor that can influence the variation in sperm size is the epididymal environment as sperm morphometry has shown to vary throughout the epididymal regions (Gutiérrez-Reinoso et al., 2016), possibly because of fluid reabsorption, hence increased osmolality. In cats, for instance, reduced within-male variation in sperm head ellipticity is related to higher epididymal mass and sperm concentration (Pintus et al., 2021). In agreement with these findings, we found that low intramale CV in sperm head ellipticity was associated with higher sperm concentration in the epididymal tails of common elands, a finding that may support the role of the epididymal environment in determining a more uniform sperm population.

Overall, the positive relationship between male secondary sexual characters and spermatogenic function found in this study provides support, at least to some extent, to the fertility-linked hypothesis, which predicts that male phenotype covaries with functional fertility (Sheldon, 1994). In contrast, sperm competition games assume a trade-off between pre- and post-copulatory investment (Parker et al., 2013). Across the animal kingdom, evidence has been found to support each or none of these hypotheses (reviewed by Simmons et al., 2017). A complex variety of factors may affect the slope of relationships including genetic variation, mating strategy or environmental conditions. In mammals, for instance, the slope of the relationship between primary and secondary sexual characters can be influenced by the mating strategy, as this trade-off is prominent in species in which weaponry investment is effective in female monopolization (Lüpold et al., 2014). Common elands usually live in large groups (up to 500 animals) that are often composed of mixed-sex herds of one to four adult males and up to more than 50 females (Pappas, 2002; Bro-Jørgensen et al., 2015). Under such mating system in which males likely fail in female monopolization, it would be more advantageous for males to equally invest their limited resources both in pre- and post-copulatory sexual traits. This would contribute to explain the positive relationship between horn size and testicular function found in this study. Another aspect to consider is that in many sexually dimorphic ungulates, secondary sexual characters like horns or antlers can simultaneously serve both as armaments and ornaments. By contrast, in a comparative study in primate species, Lüpold et al. (2019) found that only those traits that play a role as sexual ornaments trade-off against testes size in contrast to those regarded as weapons. In the light of our findings, future research effort should be directed towards the understanding of other complex signaling traits of male elands such as pelage ornament (e.g., frontal hairbrush size), dewlap droop, and the peculiar sounds presumably produced by the carpal joints, known as knee-clicks (Bro-Jørgensen and Dabelsteen, 2008; Bro-Jørgensen and Beston, 2015). Another aspect to bear in mind is that, under our experimental conditions, optimal nutritional intake was guaranteed to all animals both in terms of quantity and quality, which may mask any potential trade-off between somatic and testicular investment (Parker, 2016). As previously observed in fallow deer, nutrition has a clear impact on spermatogenic function and sperm morphometry during the sexual development (Ros-Santaella et al., 2019). It remains therefore to be tested whether under restricted access to nutritional resources a positive relationship would be still found between primary and secondary sexual traits’ development. It is also important to highlight that our study was performed on a captive population of common eland. On one hand, this experimental design allowed us to explore the relationship between the development of primary and secondary sexual characters under controlled environmental conditions, but on the other hand, it limits the extrapolation of our findings to natural populations.

The mean horn length found in our study agrees with data reported by previous authors in the adult male common eland (Bro-Jørgensen, 2007; Bro-Jørgensen and Dabelsteen, 2008), which provides support that final horn length is achieved relatively early during the male life and decreases through adulthood because of wearing (Jeffery and Hanks, 1981). Thus, adverse conditions experienced during early development may affect not only the ongoing growth of the individual but also its sexual attractiveness during adulthood (Lindström, 1999). Although age is intrinsically linked to sexual development and underlie the relationships between primary and secondary sexual characters, an unexpected finding of this study was that horn size is not related neither to the spermatic index nor to the percentage of sperm cells with normal morphology, which typically increase throughout this phase of male reproductive life (Rajak et al., 2014; Brito, 2021). Nevertheless, both parameters did not differ between common elands in early and late stages of post-pubertal development. Among the traits employed in this study to assess the horn development, it is remarkable to note that the spiral length showed the strongest correlation with the Sertoli cell efficiency. In addition, we also found that horn basal circumference was significantly associated with intramale variation in sperm head size and testicular investment in the seminiferous tubular and epithelial areas. The spiral length and the basal circumference are the horn traits responsible for the sexual dimorphism in common elands as males show horns with more pronounced spirals and greater basal circumference than females (Jeffery and Hanks, 1981; Pappas, 2002). The spiral ridge of the eland horn core has shown different mechanical and chemical properties compared to other parts of this permanent appendage, which may avoid fractures during male-male contests (Cappelli et al., 2018). The spiral of the horn, as bumps and ridges on the horns of various antelopes and gazelles, serve indeed to hold these appendixes together during the match, allowing the opponents to develop full strength wrestling engagement (Geist, 1966). In a comparative study across 104 bovid species, Caro et al. (2003) found that, at least in females, twisted horns are associated with wrestling behavior. In the same study, the authors also found that the outward-facing and twisted horns of bovids are also associated with polygynous and large mating groups. Such finding was later supported by Bro-Jørgensen (2007) who found that in polygynous mating system, in which sexual selection is likely to be strong, horn shape is more variable and elaborate than in monogamous species, in which horns tend to be simple and straight. Taken together, male elands with more developed spirals and greater basal circumference may signal not only their fighting ability but also their reproductive competence (i.e., spermatogenic efficiency and sperm size homogeneity). Another non-mutually exclusive hypothesis is that increased horn size may provide a better mechanism for thermoregulation, which may also have beneficial effect on spermatogenesis. Within the thermal neutral zone, the common eland metabolism is estimated to be around 30% higher than that of Hereford cattle, a bovid species of comparable size (Taylor and Lyman, 1967), although other authors did not find support to this finding (Kotrba et al., 2007). Spermatogenesis requires a cool environment (usually 2°C–6°C below the body core temperature), which is normally guaranteed by the scrotal sac and the complex anatomical structures associated with the latter (i.e., pampiniform plexus, dartos, cremaster, and sweat glands). Because these complex anatomical structures develop with age and influence sperm motility and morphology (Brito et al., 2012), their role is likely more critical in young individuals because of their higher metabolism compared to that of adults. In line with our hypothesis, in the male Alpine ibex (*Capra ibex*), the early development of horns is a suitable predictor of individual reproductive success later in life, whereas the late development of this secondary sexual trait did not seem to significantly relate to it (Willisch et al., 2015). In addition, like other large herbivores adapted to xeric environment (Hetem et al., 2016), common elands can employ adaptive heterothermy by increasing their body temperature in response to high environmental heat load to reduce the water loss (Taylor and Lyman, 1967; but see also Fuller et al., 2004). Because horn size and shape can provide a more efficient mechanism for heat loss (Picard et al., 1994; 1999), and the latter can be beneficial for the spermatogenesis, we can speculate that bigger horn size can provide a more efficient mechanism not only for the body but, indirectly, also for the testicular thermoregulation. On the other hand, no direct link between horn asymmetry and reproductive function was found, probably because of the absence of environmental stressors in our animal population. Using a large dataset, Chirichella et al. (2020) found that environmental stressors (e.g., snow cover duration, population density) experienced in early life influence the symmetrical horn development in the Alpine chamois (*Rupicapra rupicapra*).

In conclusion, greater male investment in secondary sexual characters is related to enhanced spermatogenic function and increased homogeneity in sperm cell dimension. During the sexual development, common elands that display bigger horns show increased testes size, spermatogenic investment, Sertoli cell efficiency and reduced intramale variation in sperm head size and shape. The findings from this study increase our understanding about the role of secondary sexual traits that, beyond sexual selection dynamics, might be involved in the thermoregulatory mechanisms that guarantee the optimal conditions for sperm production.

## Data Availability

The original contributions presented in the study are included in the article/Supplementary Material, further inquiries can be directed to the corresponding authors.

## Supplementary material

SUPPLEMENTARY TABLE S1 Principal component analysis of horn size, Sertoli cell efficiency, and intramale variation in sperm head size in ≤30 months old and >30 months old post-pubertal common elands.

SUPPLEMENTARY TABLE S2 Dataset of male post-pubertal common elands.
