# Supplementary material for: Horn size is linked to Sertoli cell efficiency and sperm size homogeneity during sexual development in common eland (Taurotragus oryx)
Source: Front Cell Dev Biol. 2024 Aug 20;12:1421634. doi: 10.3389/fcell.2024.1421634 (PMC11368866; doi:10.3389/fcell.2024.1421634)
Supplement: Supplementary file 2 [file Table1.docx]

**Supplementary Table 1. Principal component analysis of horn size, Sertoli cell efficiency, and intramale variation in sperm head size in** **≤30 months old and >30 months old post-pubertal common elands**

|  | PC1  ≤30 months old | *p* | PC1  >30 months old | *p* |
| --- | --- | --- | --- | --- |
| *Horn size* |  |  |  |  |
| Horn length | 0.896 | <0.001 | 0.974 | <0.001 |
| Spiral length | 0.975 | <0.001 | 0.965 | <0.001 |
| Basal circumference | 0.460 | 0.114 | 0.943 | <0.001 |
| Eigenvalue | 1.97 |  | 2.77 |  |
| Variance explained (%) | 65.52 |  | 92.34 |  |
| KMO test | 0.376 |  | 0.751 |  |
| Bartlett’s test | <0.001 |  | <0.001 |  |
|  |  |  |  |  |
| *Sertoli cell efficiency* |  |  |  |  |
| log SEI | -0.997 | <0.001 | -0.993 | <0.001 |
| log SSEI | 0.949 | <0.001 | 0.972 | <0.001 |
| log RS/SC | 0.980 | <0.001 | 0.990 | <0.001 |
| log ES/SC | 0.953 | <0.001 | 0.960 | <0.001 |
| log GC/SC | 0.999 | <0.001 | 0.997 | <0.001 |
| Eigenvalue | 4.76 |  | 4.83 |  |
| Variance explained (%) | 95.20 |  | 96.60 |  |
| KMO test | 0.649 |  | 0.723 |  |
| Bartlett’s test | <0.001 |  | <0.001 |  |
|  |  |  |  |  |
| *Intramale variation in sperm head size* |  |  |  |  |
| log head width CV | 0.837 | 0.002 | 0.989 | <0.001 |
| Head area CV | 0.928 | <0.001 | 0.939 | 0.002 |
| Head ellipticity CV | 0.908 | <0.001 | 0.921 | 0.003 |
| Eigenvalue | 2.39 |  | 2.71 |  |
| Variance explained (%) | 79.51 |  | 90.30 |  |
| KMO test | 0.699 |  | 0.572 |  |
| Bartlett’s test | 0.008 |  | 0.001 |  |

CV: coefficient of variation; KMO: Keiser-Meyer-Olkin; SEI: Sertoli cell index; SSEI: spermatozoa-Sertoli cell index; RS/SC: ratio of round spermatids to Sertoli cells; ES/SC: ratio of elongated spermatids to Sertoli cells; GC/SC: ratio of germ cells to Sertoli cells. PC: Principal component.
